# Efficacy and safety of antiangiogenic agents combined with HIFU in the treatment of advanced liver cancer

**DOI:** 10.3389/fonc.2025.1652434

**Published:** 2025-09-26

**Authors:** Rong Wu, Qiuling Shi, Dongbiao Liao, Yao Liao, Feng Sun, Yu He, Peiyang Mao, Lingli Fan, Yuxi Bai, Gang Feng

**Affiliations:** ^1^ Department of Oncology, Mianyang Central Hospital, Mianyang, Sichuan, China; ^2^ School of Public Health, Chongqing Medical University, Chongqing, China; ^3^ Department of Radiology, Mianyang Central Hospital, Mianyang, Sichuan, China

**Keywords:** liver cancer, antiangiogenic agents, high-intensity focused ultrasound, ultrasound grayscale, coagulative necrosis

## Abstract

**Background:**

Although antiangiogenic agents and HIFU (High-intensity focused ultrasound) are extensively used in the systematic treatment of advanced primary and secondary liver cancer, respectively, the efficacy and safety of their combination remain unclear. Thus, this study aimed to investigate the efficacy, safety, and synergistic effect of the combination of antiangiogenic drugs with HIFU in the treatment of advanced liver cancer.

**Methods:**

Advanced liver cancer patients undergoing HIFU were included and matched 1:1 to two groups based on admission criteria: patients who received HIFU combined with antiangiogenic agents were assigned to the Combined HIFU group, whereas those who received HIFU but not antiangiogenic agents were assigned to the Only HIFU group. Then, therapeutic parameters, short-term efficacy, long-term survival, and safety of HIFU were analyzed and compared in this study.

**Results:**

There were 25 cases in both the Combined HIFU and Only HIFU groups. A significant difference was noted in the median ultrasound grayscale (hyperechoic region) occurrence time between the two groups (*p=0.04*). The coagulative necrosis rate, ORR, and DCR of liver lesions were numerically higher in the Combined HIFU group (60%, 64%, and 96%) than those in the Only HIFU group (44%, 36%, and 84%). Contrastingly, mOS did not differ significantly between the two groups. (HR,0.91; 95% CI, 0.45 to 1.82; *p* = 0.79). Finally, Acute adverse events (AEs) primarily included skin-burning pain, fever, and impaired liver function, and the incidence of infectious fever and impaired liver function was lower in the combined HIFU group.

**Conclusion:**

Antiangiogenic agents combined with HIFU are effective and safe in the treatment of advanced primary and secondary liver cancer.

## 1 Introduction

According to epidemiological studies (1), the mortality rate of liver cancer ranks the third in the world, and the incidence rate is increasing year by year. In China, 78.6% of liver cancer cases are diagnosed at the advanced stage (2), with the liver being the most common metastatic site for malignant solid tumors. At present, anti-angiogenic agents have been widely used in the systemic treatment of advanced primary liver cancer and secondary liver cancer. When local lesions are poorly controlled, intensive treatment combined with local interventions is often required. Primary therapeutic approaches for liver cancer encompass surgery, Transcatheter Arterial Chemoembolization (TACE), radiofrequency ablation, and radiotherapy. However, the first three methods are invasive, and antiangiogenic agents must be discontinued for at least 4 weeks prior to treatment. This may lead to tumor progression and missing the optimal window for local treatment (3). While radiation therapy is non-invasive, it is associated with prolonged treatment duration, high costs, and substantial variability in treatment equipment and technology across different centers. High-intensity focused ultrasound (HIFU) is a completely non-invasive local thermal ablation therapy that offers numerous advantages, including high real-time guidance accuracy, short treatment time, fast recovery, and low cost. Theoretically, it can be simultaneously performed with antiangiogenic agents, eliminating the need for discontinuation of antiangiogenic agents before treatment. Notwithstanding, the efficacy and safety of this combination remain elusive. At the same time, liver cancer tumor cells are characterized by a rich blood supply that dissipates heat and complicates HIFU treatment. Considering that anti-angiogenic agents can inhibit tumor neovascularization, as well as reduce vascular permeability and blood supply, their combination with HIFU may potentially enhance treatment efficiency and concurrently shorten operation time. Therefore, this study aimed to explore the efficacy, safety, and synergistic efficiency of the combination of antiangiogenic agents with HIFU in the treatment of advanced liver cancer.

## 2 Materials and methods

### 2.1 Study design

This study employed a case-control design. The experimental cohort comprised 25 patients with advanced liver cancer who underwent HIFU therapy subsequent to antiangiogenic targeted therapy at Mianyang Central Hospital, China, between January 5, 2022, and June 19, 2024. Using SPSS 22.0 (IBM Corp.), matched controls were selected for each case from the pool of advanced liver cancer patients who did not receive antiangiogenic targeted therapy. Matching was performed based on three key variables: liver cancer subtype, liver function status, and size of the target lesion with a 1:1 matching ratio. Ultimately, 25 eligible matched controls were successfully enrolled for subsequent analyses.

### 2.2 Subjects

Patients with advanced liver cancer (defined as stage III-IV primary liver cancer and secondary liver cancer) undergoing HIFU between January 5,2022 and June 19,2024were eligible for inclusion. All patients were staged according to AJCC eighth edition TNM staging criteria (4), and the diagnosis was confirmed by pathological examination. The inclusion criteria were as follows: patients with liver lesions who are not eligible or unwilling to undergo surgery, radiotherapy, TACE, and radiofrequency ablation with well-controlled non-liver lesions. The exclusion criteria were as follows: having previously received HIFU, other local treatment, or less than one treatment cycle of antiangiogenic agent targeted therapy, incomplete or unavailable HIFU treatment parameters and follow-up data, including but not limited to postoperative monitoring of signs and symptoms, laboratory test results, and liver contrast-enhanced MRI (CE-MRI) before and within 1 month after surgery. Patients in the experimental group who were administered antiangiogenic agents at the time of HIFU treatment were assigned to the Combined HIFU group, whereas the Only HIFU (control) group comprised patients who did not receive antiangiogenic agents before HIFU treatment.

### 2.3 HIFU therapeutic procedure

Patients were treated using a high-intensity focused ultrasound treatment system (Model: HIFU-JC200, Chongqing Haifu Medical Technology, Chongqing, China) equipped with a real-time ultrasound guidance device. The detailed information about the HIFU treatment is as follows.

#### 2.3.1 Patients preparation

All patients signed an informed consent prior to HIFU and consumed a diet low in liquid residue for 3 days before treatment. Patients were kept under fasting conditions 12 hours before surgery, and compound polyethylene glycol electrolyte powder was orally administered to induce diarrhea. Additionally, an enema was performed before surgery. Under general anesthesia, the patient’s skin was degassed and degreased after tracheal intubation, and a catheter was inserted to minimize patient discomfort. For patients with lesions in the left liver, a nasogastric tube was inserted to decompress the stomach, improve visualization of liver lesions, and minimize the risk of gastric heat damage.

#### 2.3.2 Treatment position

the lesions in the right lobe of the liver were treated in the right lateral position, while the lesions in the left lobe of the liver were treated in the prone position.

#### 2.3.3 Selection of ultrasound path

there should be no gas, scar, bone, or calcification lesions in the ultrasound path that had strong reflection effects on ultrasound.

#### 2.3.4 Pre-scan

the treatment range was determined based on the target lesion and its proximity to important organs. The layer spacing was generally set at 3mm or 5mm.

#### 2.3.5 The treatment probe was generally selected at 0.8 MHz

Transducers with focal lengths of 115mm and 165mm were often used based on the depth of tumor treatment. The scanning mode was mainly linear scanning; the scanning direction was usually longitudinal scanning, and “θ” was set to 90 degrees. The treatment approach was carried out from the deeper layer to the superficial layer of the target lesion.

### 2.4 Outcome

#### 2.4.1 Primary endpoint

the objective response rate (ORR), as assessed by the investigators, was determined in accordance with the modified Response Evaluation Criteria in Solid Tumors version 1.1 (mRECIST 1.1). ORR was defined as the composite of complete remission (CR) and partial remission (PR) of the tumor following treatment. The details were as follows: complete response (CR): complete coagulative necrosis of the target lesion; partial response (PR): the longest diameter of the coagulated necrosis area of the target lesion was ≥ 30% of the baseline; stable disease (SD): no PR and no PD; disease progression (PD): no coagulative necrosis of the target lesion and at least a 20% increase in the longest diameter.

In coagulative necrotic lesions, intracellular proteins undergo denaturation, organelles are disrupted, and the integrity of cell membranes is lost-resulting in irreversible cellular inactivation and the complete absence of tumor cell viability. Clinically, this therapeutic effect is equivalent to lesion regression. Therefore, the inclusion of “coagulative necrosis” in the RECIST 1.1 criteria was scientifically justified.

#### 2.4.2 Secondary endpoint

I. Ultrasound grayscale occurrence time: defined as the time point at which the hyperechoic region first appears on grayscale images during real-time ultrasound monitoring of HIFU therapy.

The temporal dynamics of grayscale changes, including the onset speed, expansion rate, and temporal stability of hyperechoic regions, can effectively reflect the efficiency of thermal energy deposition and the tissue response to HIFU. Thus, this parameter served as a valuable reference indicator for dynamically assessing therapeutic efficacy and guiding the adjustment of treatment intensity intraoperatively.

II. Investigator-assessed disease control rate (DCR): defined as the sum of tumor responses achieving remission CR and PR and SD following treatment.

III. Overall survival (OS): defined as the time from the initiation of planned HIFU treatment to death of the patient from any cause.

IV. Acute adverse events (AEs): it was examined based on patients’ symptoms and signs within 3 days post-treatment and liver function 24 h after surgery. Pain was evaluated using the numeric rating scale (NRS), whilst liver function was evaluated using the Common Adverse Reactions Scale 5.0 (CTCAE5.0).

### 2.5 Statistical analysis

The Kolmogorov-Smirnov statistical test was performed to determine the normality of the data. Non-normally distributed data were presented as medians (P25, P75) and compared using the Wilcoxon rank sum test. The chi-square test was used to compare gender, liver cancer type, and liver function grade. The median follow-up time was calculated using the Reverse Kaplan-Meier method. Kaplan-Meier survival analysis was performed to plot patient survival curves and calculate the median survival period. COX regression analysis was utilized to calculate the risk ratio (HR) for survival outcomes. IBM SPSS 22.0 statistical software was used for statistical analysis and *P*<0.05 was considered statistically significant.

## 3 Results

**Figure 1:**
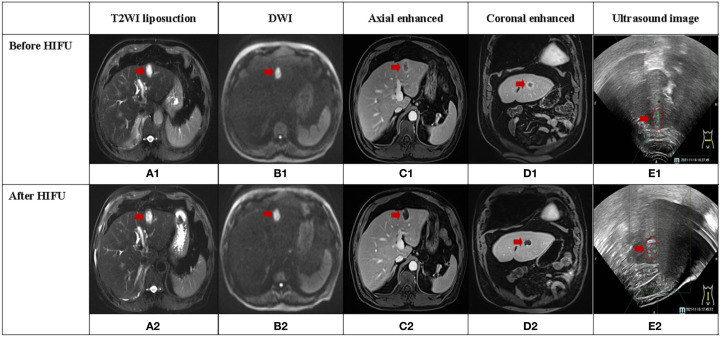
A case of secondary liver cancer achieved complete response (CR) after HIFU treatment, and the lesion was located in the left lobe of the liver (red arrow). **(A1-D1, A2-D2)** illustrate MRI sequences before and after HIFU treatment, respectively. **(E1, E2)** display ultrasound images before and after HIFU treatment, respectively. **(E2)** depicts significant grayscale changes in the lesion.

**Figure 2:**
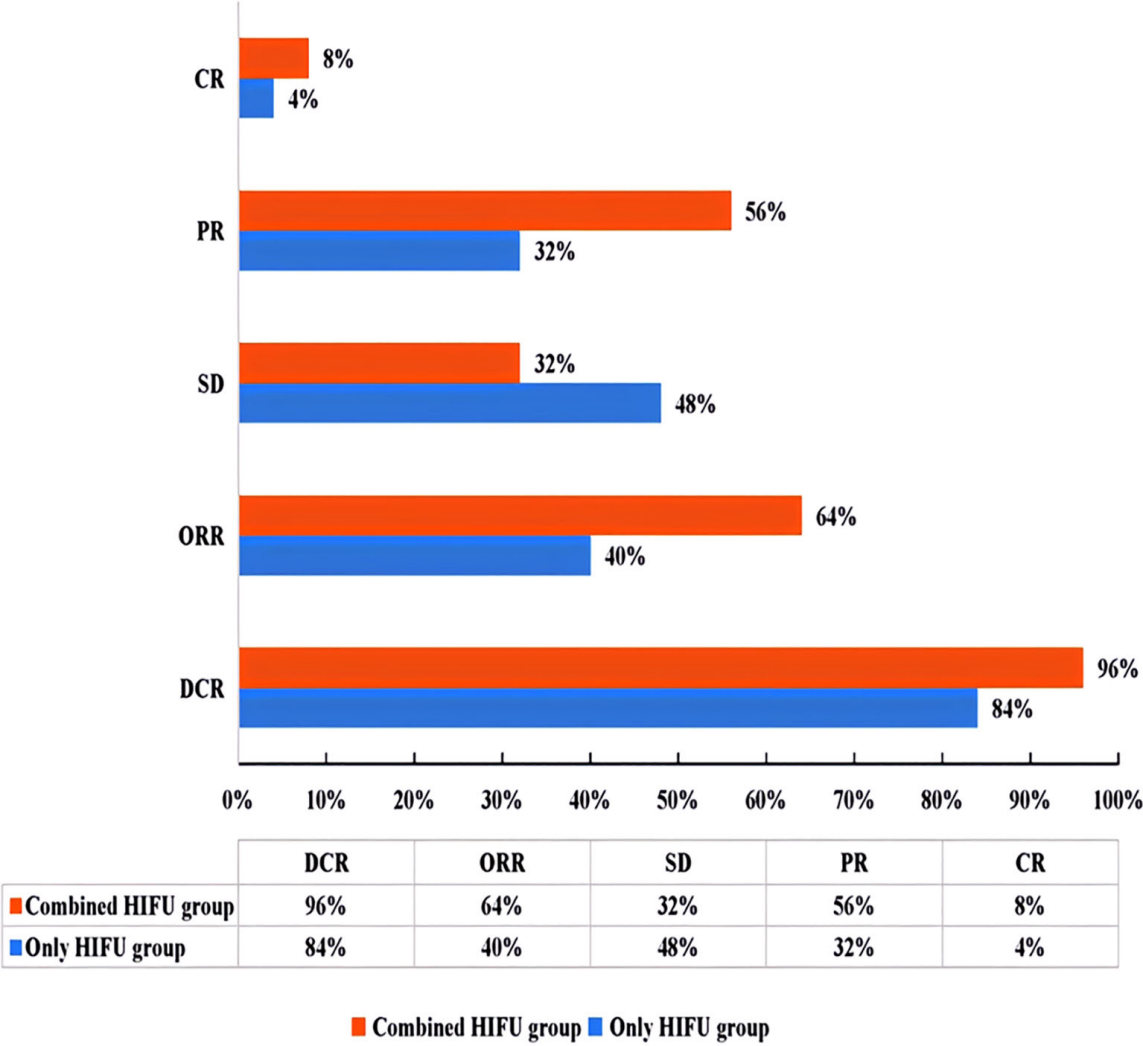
Efficacy evaluation chart according to mRECIST1.1 criteria (ORR=CR+PR; DCR=CR+PR+SD). ORR, objective response rate; DCR, disease control rate; CR, complete response; PR, partial response; SD, stable disease.

In the Combined HIFU group, there were 12 male and 11 female patients with a median age of 63 years. In the Only HIFU group, there were 19 male and 6 female patients with a median age of 60 years. With respect to the type of liver cancer, each group included 9 cases of primary liver cancer and 16 cases of secondary liver cancer. Among the primary liver cancer cases, two-thirds were at stage III and one-third at stage IV; all secondary liver cancer cases were at stage IV. No patients in either group had Child-Pugh class C liver function. There were no statistically significant differences in baseline characteristics between the two groups (p>0.05), indicating that they were comparable. In the Combined HIFU group, the mean number of cycles of antiangiogenic targeted therapy administered prior to HIFU treatment was 4.5 (range:1–11). Specifically, 60% of the patients received bevacizumab, 24% lenvatinib, 12% anlotinib, and 4% fruquintinib (**Table 1**). HIFU treatment parameters of the two groups are presented in **Table 2**. During HIFU treatment, changes in ultrasound grayscale images can reflect real-time treatment efficacy, corresponding to the degree of coagulated necrosis in target lesions (5–7). As illustrated in **Figure 1**, the intraoperative ultrasound image displayed significant alterations in grayscale in a lesion in the left lobe of the liver after HIFU treatment, with the echo intensity being significantly higher than the initial treatment image. In the current study, the median grayscale occurrence time was 25s in the Combined HIFU group and 104s in the Only HIFU group, and the difference was statistically different (z=-2.00, *p=0.04*). This finding suggests that the combination treatment achieved faster and more sensitive treatment responses. Under the guidance of a senior radiologist, CE-MRI images were analyzed, and changes in lesions before and after HIFU treatment were compared, measuring lesion size and coagulated necrosis area to determine treatment efficacy according to mRECIST1.1. 60% (15/25) of patients in the Combined HIFU group had a higher degree of coagulative necrosis compared to the Only HIFU group, and the 1-month disease control rate (DCR) was 96% and 84%, respectively. Despite some lesions not displaying evidence of coagulative necrosis, follow-up revealed that they benefited from HIFU treatment in controlling short-term lesion progression, and the likelihood of local lesion progression after treatment was only 1/4 of compared to the control group (**Figure 2**, **Table 3**). Furthermore, this study also demonstrated that the efficacy of HIFU therapy varied across different subtypes of advanced carcinoma. Both the coagulative necrosis rate and DCR were higher in patients with primary liver cancer than in those with secondary liver cancer, with this difference being more pronounced in the combined therapy group (**Table 3**). However, due to the small sample size of each subgroup, statistical analysis could not be performed. Therefore, studies with a larger sample size will be required to further validate this finding.

**Table 1:** Baseline characteristics of patients.

Patient demographics	Combined HIFU group	Only HIFU group	χ^2^ /z, *p*
Number of patients (n)	25	25	
Gender (n, %)			2.23, 0.14
Male	14 (56)	19 (76)	
Female	11 (44)	6 (24)	
Age (years) Median (P_25,_ P_75_)	63 (51.50,70.50)	60 (52.00,69.50)	-0.19, 0.85
Liver cancer type (n, %)			0.00, 1.00
Primary	9 (36)	9 (36)	
Secondary	16 (64)	16 (64)	
Child-Pugh classification of liver function (n, %)			0.94,0.33
A	20 (80)	17 (68)	
B	5 (20)	8 (32)	
C	0 (0)	0 (0)	
Antiangiogenic agents (n, %)			
Bevacizumab	15(60)		
Lenvatinib	6 (24)		
Anlotinib	3 (12)		
Fruquintinib	1 (4)		
Treatment cycles	4.5 (1∼11)		

**Table 2:** HIFU treatment parameters between the combined HIFU group and the Only HIFU group.

HIFU treatment parameters: Median (P_25,_ P_75_)	Combined HIFU group	Only HIFU group	z, *p*
Mean treatment power (w)	400.00 (350.50,400.00)	379.00 (342.00, 397.00)	-1.78, 0.08
Irradiation time (s)	1664.00 (880.50,2122.00)	1472.00(686.00,2102.00)	-0.83, 0.41
Treatment intensity^*^(s/h)	605.00 (514.50,686.50)	606.00 (553.50, 646.00)	-0.17, 0.87
Treatment dose (J)	652550.00(312800.00,835050.00)	557550.00(230700.00,751650.00)	-0.86, 0.39
Treatment volume (cc)	5.63 (3.79,7.65)	4.28 (2.21, 7.58)	-1.04, 0.30
Work done^#^ (J)	652386.00(312615.00, 834776.50)	557740.00(231900.00,752095.00)	-1.08, 0.28
Grayscale occurrence time: median (s)	25.00 (4.00, 190.50)	104.00 (25.50, 399.50)	-2.00, 0.04

*Treatment intensity = irradiation time/treatment time, ^#^Work done = mean treatment power × irradiation time.

**Table 3:** Treatment outcomes for patients in the combined HIFU and Only HIFU groups.

Liver cancer type	Combined HIFU group (n,%)			Only HIFU group (n,%)		
	Primary (n=9)	Secondary (n=16)	Total (n=25)	Primary (n=9)	Secondary (n=16)	Total (n=25)
Lesion coagulative necrosis						
Yes	7(77.78)	8(50.00)	15 (60.00)	6(66.67)	5(31.25)	11 (44.00)
No	2(22.22)	8(50.00)	10 (40.00)	3(33.33)	11(68.75)	14 (56.00)
mRECIST1.1						
CR	1(11.11)	1(6.25)	2 (8.00)	0(0.00)	1(6.25)	1 (4.00)
PR	5(55.56)	9(56.25)	14 (56.00)	4(44.44)	4(25.00)	8 (32.00)
SD	3(33.33)	5(31.25)	8 (32.00)	4(44.44)	8(50.00)	12 (48.00)
PD	0(0.00)	1(6.25)	1 (4.00)	1(11.11)	3(18.75)	4 (16.00)

**Figure 1** depicts CE-MRI and intraoperative ultrasound images of a CR secondary liver cancer before and after HIFU treatment. The lesion was located in the left outer lobe of the liver and measured approximately 3cm×2cm. Pre-treatment revealed a low signal on T1WI, a high signal on T2WI, a high signal on DWI, and uneven enhancement on contrast-enhanced scan, predominantly with marginal ring enhancement. Post-treatment imaging showed a low signal on T1WI, a high signal on T2WI, and a high signal on DWI. There was no evidence of enhancement areas on enhanced scan, only marginal linear enhancement. Before and after treatment, coagulative necrosis (non-enhanced area) completely covered the original lesion. Ultrasound images displayed hyperechoic grayscale change (hyperechoic region) after treatment, which was consistent with the coagulated necrosis area observed on CE-MRI images.

**Figure 3:**
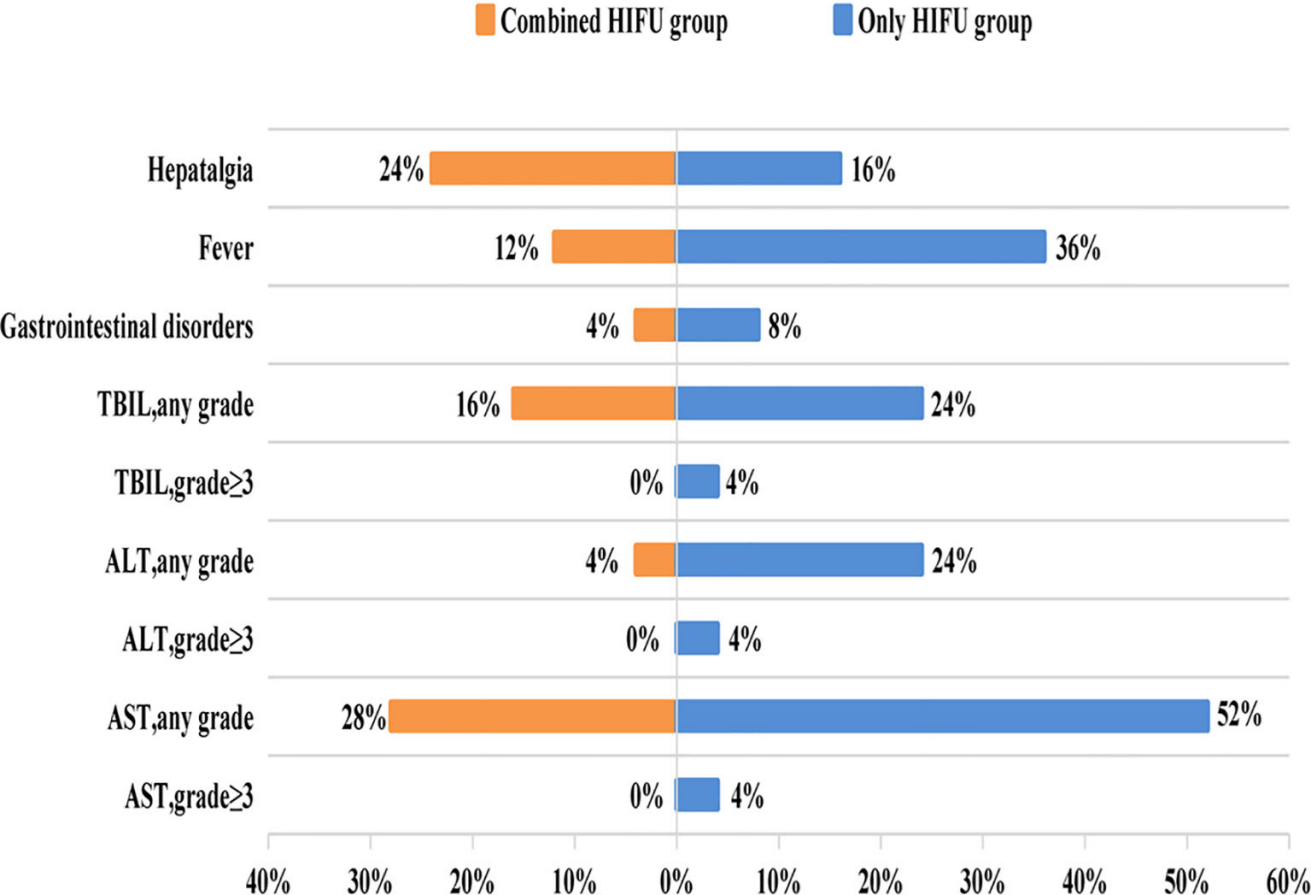
Bidirectional bar accumulation diagram of treatment-emergent adverse event.

No skin infection, tumor rupture, hemorrhage, or organ perforation occurred in either group. 20% (10/50) of patients developed hepatalgia and mild to moderate burning pain on the skin, with no patients experiencing severe pain. Mild pain was relieved after treatment with ice and non-steroidal anti-inflammatory drugs, and moderate pain in two patients was alleviated after treatment with weak opioid painkillers and ice. 24% (12/50) of patients were febrile within 3 days after the operation, largely due to bile duct infection and sepsis, with the incidence of fever being 3 times in the Only HIFU group compared with the Combined HIFU group. All liver function injuries occurred within 24h after HIFU, with most of them being transient and resolving without treatment. Notably, no patients developed grade 3 or higher liver damage in the Combined HIFU group, whereas 12% developed grade 3 or higher liver damage in the Only HIFU group (**Table 4**, **Figure 3**).

**Table 4:** TEAEs in the study population.

	Combined HIFU group (n, %)	Only HIFU group (n, %)
Hepatalgia^@^	6 (24)	4 (16)
Mild	5 (20)	3 (12)
Moderate	1 (4)	1 (4)
Severe	0 (0)	0 (0)
Fever	3 (12)	9 (36)
Non-infective	1 (4)	5 (20)
Infective	2 (8)	4 (16)
Gastrointestinal disorders	1 (4)	2 (8)
Liver function^&^		
TBIL	4 (16)	6(24)
Any grade	0 (0)	1(4)
Grade≥3		
ALT	1 (4)	6 (24)
Any grade	0 (0)	1 (4)
Grade≥3		
AST	7 (28)	13 (52)
Any grade	0 (0)	1 (4)
grade≥3		

^@^Hepatalgia: numeric rating scale (NRS); ^&^liver function: common Adverse reactions Scale 5.0 (CTCAE5.0). Abbreviations: TEAE, treatment-emergent adverse event; TBIL, total bilirubin; ALT, alanine aminotransferase; AST, aspartate aminotransaminase.

**Figure 4:**
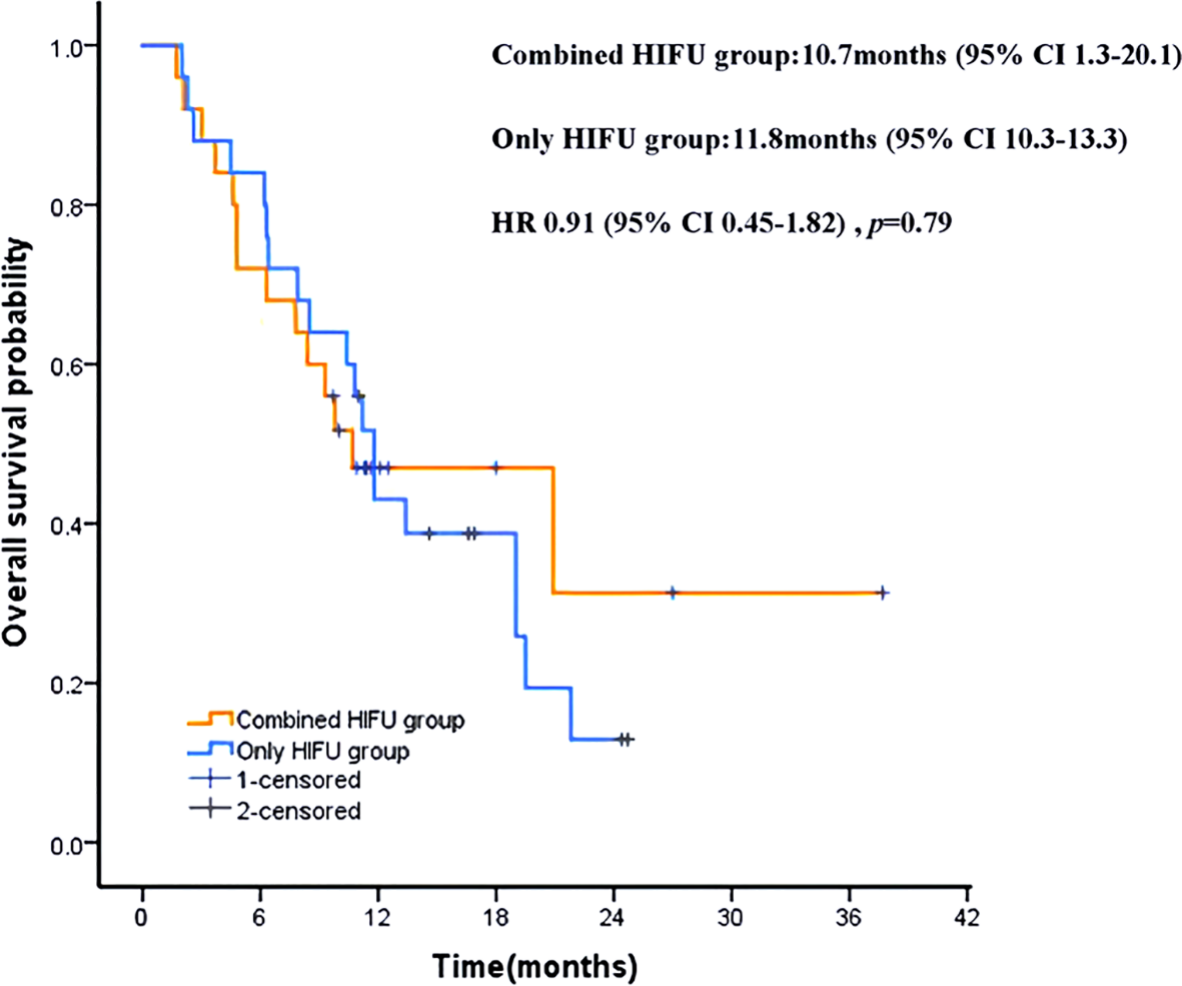
Kaplan-Meier patient survival curves. HRs were derived from stratified Cox proportional hazards models. HR, hazard ratio.

The median follow-up was 16.9 months (95% CI 12.1-21.7). Until the end of the follow-up period, 11 patients (44%) in the Combined HIFU group survived, with a 12 months survival rate of 48% and median overall survival (mOS) of 10.7 months (95% CI 1.3-20.1). On the other hand, six patients (24%) in the Only HIFU group survived, with a 12-month survival rate of 44% and an mOS of 11.8 months (95% CI 10.3-13.3). There was no significant difference in mOS between the two groups (HR, 0.91; 95% CI, 0.45 to 1.82; *p* = 0.79) (**Figure 4**). All deaths were cancer-related.

## 4 Discussion

As is well documented, the liver is one of the organs with the most common incidence of malignant tumor (8). Hepatocellular carcinoma (HCC) is the main pathological type of primary liver cancer, accounting for approximately 75% to 85%, which is one of the malignant tumors with the highest morbidity and mortality globally (9). China ranks fourth in liver cancer morbidity and second in mortality. Moreover, patients are often diagnosed at advanced stages, contributing to the poor prognosis of liver cancer patients (10). In addition, HCC is highly malignant and prone to recurrence and metastasis, which brings challenges to treatment (1, 11–13). Secondary liver cancer is also referred to as metastatic liver cancer. The dual blood supply of the liver results in an abnormally rich blood flow. Consequently, upon entering the bloodstream, cancer cells can move to the liver to form metastases. The actual incidence rate of liver cancer is currently not precisely known, but for most common malignant tumors, such as those in the digestive tract, breast cancer, and prostate cancer, the liver is the most frequently affected organ, metastatic liver cancer is more common than primary liver cancer (14).This is largely consistent with what has been reported in this study. Among patients with advanced liver cancer, 64% are diagnosed with metastatic liver cancer, and 53.1% of these metastatic cases originate from the colorectum. The median survival time of patients with untreated liver metastases is merely 6.9 months (15), making it the leading cause of death in patients with CRC (16, 17). Therefore, active treatment of liver metastases is an effective strategy for delaying disease progression and improving survival outcomes.

Tumor pathogenesis is intricately related to vascular diseases, with the ligand and receptor families of vascular endothelial growth factor (VEGF) playing a decisive role. By actively targeting VEGF, antiangiogenic agents inhibit tumor neovascularization and promote the normalization of abnormal blood vessels to eventually inhibit tumor proliferation. The FDA has approved over a dozen drugs targeting the VEGF/VEGFR pathway, including bevacizumab, lenvatinib, fuquitinib, and anlotinib, which have been used for the treatment of a range of cancers (18), encompassing primary liver cancer (19, 20), CRC (21, 22), lung cancer (23, 24), ovarian cancer (25), and soft tissue sarcoma (26). Rajabi et al. (27) pointed out that effective anti-angiogenic therapy prevents tumor growth. Nonetheless, under specific conditions, they cannot eradicate tumors with single-agent therapies due to compensatory mechanisms, necessitating combination therapies to enhance efficacy. Of note, local treatment can achieve optimal anti-tumorigenic effects in patients with poor local lesion control.

HIFU is a non-invasive technique that employs focused high-energy ultrasound (0.8-3.5MHz) directed at local lesions, thereby instantaneously increasing the temperature at the focal point within seconds and causing irreversible coagulative necrosis of tumor lesions without damaging surrounding tissues (28–30). HIFU can also use (boiling) tissue fragmentation to generate non-thermal effects and destroy focal tissues (31). Compared with surgical interventions and other ablative procedures, the primary advantage of HIFU is its non-invasive nature, which mitigates the risk of tumor spread and metastasis caused by invasive procedures. Furthermore, HIFU allows for the use of antiangiogenic agents without the need for discontinuation before invasive treatment, ensuring that patients do not miss the optimal treatment window and optimizing therapeutic outcomes. HIFU has been widely used in the treatment of both benign and malignant tumors (30, 32, 33–36). Prachee et al. (37) concluded that, to date, HIFU remains the only completely non-invasive local treatment for liver malignant tumors. Herein, no severe adverse events such as skin infection and necrosis, tumor rupture and bleeding, organ perforation, fistula formation, or death were recorded after HIFU treatment for advanced primary liver cancer or secondary liver cancer, validating its non-invasive nature and safety profile. At the same time, HIFU is not limited by the size of the lesion. Wu et al. (38) documented that HIFU can safely and effectively be used for the treatment of large liver cancers with diameters exceeding 4–14 cm. In the present study, 54% (27/50) of lesions had a longest straight length greater than 5 cm. Although other ablation techniques (e.g., radiofrequency ablation, laser ablation, microwave ablation, and cryoablation) are also minimally invasive, they cannot effectively ablate liver cancer with a diameter exceeding 5 cm, limiting their applicability in cases of advanced liver cancers.

Magnetic resonance imaging (MRI) and ultrasound imaging (US) are currently the two mainstream modalities for guiding and monitoring clinical HIFU surgery, providing integrated treatment planning, real-time control (spatial and temperature), and evaluation (39, 40). Although MRI is highly sensitive to temperature and soft tissue contrast, it does not meet real-time imaging requirements during treatment and is costly, especially due to a lack of specialized MRI-HIFU equipment for liver cancer (41, 42). Ultrasound image-guided HIFU (US-HIFU) is the most widely used technique in the clinical setting owing to its low cost, favorable compatibility, and real-time performance (43). Thus, US-HIFU was used in this study. According to the HIFU treatment parameters in this study, a statistical difference was identified in the occurrence time of grayscale between the combined HIFU treatment group and the single HIFU group (*p=0.04*). The median grayscale occurrence time of the former was significantly shorter than that of the latter, signaling that advanced liver cancer treated with antiangiogenic agents was more sensitive to lesions and had a faster onset of effect during HIFU surgery. The grayscale of an ultrasound image is determined by the ultrasonic reflection coefficient of tissues (i.e., the intensity of reflected signals). In normal tissues, the cellular structure, density, and water content are relatively uniform, resulting in a stable reflection coefficient. Accordingly, the grayscale presents as uniform “isoechoic” (echo intensity matching the surrounding normal tissue) or “hypoechoic” (echo intensity lower than the surrounding normal tissue).After HIFU treatment, however, the proteins in the target lesion undergo denaturation. This process leads to the disruption of cellular structures, an increase in tissue density, and the formation of irregular “coagulative necrosis foci”. The density difference between the treated lesion and the surrounding normal tissue is thereby significantly enlarged, which enhances the ultrasonic reflection signals—causing the treated area to appear “hyperechoic” (echo intensity higher than the surrounding normal tissue) on ultrasound images. Therefore, the real-time monitoring of HIFU treatment efficacy can be achieved by tracking changes in ultrasonic grayscale (44).Although the irradiation time was not statistically different between the two groups, the median treatment volume in the combined HIFU group was 1 cc larger than that in the control group. Larger volumes are typically correlated with longer irradiation times. Therefore, while the total operation time was similar, larger lesions were simultaneously treated, which partially enhanced treatment efficiency, accelerated procedural workflow, and lowered the risk of intraoperative anesthesia. HIFU enables the selective destruction of tumor blood vessels with a diameter of ≤ 2 mm, while sparing major blood vessels. This mechanism blocks the nutritional supply to tumor cells, leading to their ischemic necrosis, without inducing the dissemination or metastasis of tumor cells (45).Additional studies have demonstrated that tumor vascular structure components including elastic fiber, endothelial cells all were destroyed by HIFU. After ultrasonic ablation, gray-scale of tumor nodules enhanced in ultrasonography, tumor peripheral and internal blood flow signals disappeared or significantly reduced in color Doppler flow imaging (46). In advanced liver cancer, tumors exhibit abundant blood flow and high perfusion. The heat generated by HIFU is easily dissipated by the bloodstream, leading to insufficient temperature elevation in the tumor target region. As a result, the critical threshold of an instantaneous high temperature (over 60 °C),which is required to achieve one-time coagulative necrosis, and cannot be reached rapidly. This ultimately results in limited therapeutic efficacy and prolonged treatment duration. In the present study, antiangiogenic targeted drugs are administered prior to HIFU therapy to inhibit tumor blood supply and reduce blood flow. This intervention transforms the target lesion from a hypervascular state to a hypovascular state, thereby enhancing treatment efficiency and achieving a synergistic anti-tumor effect. Currently, Some studies on HIFU combined with transcatheter arterial chemoembolization (TACE) or radiotherapy also hinges on this fundamental principle (47, 48).It is worth recognizing that this study failed to demonstrate statistical differences due to the small sample size. In the future, large randomized clinical trials are warranted to further evaluate the efficacy and safety of the combination of antiangiogenic agents with HIFU for patients with advanced liver cancer.

CE-MRI can be used to visualize coagulated necrosis of liver cancer and evaluate the effectiveness of HIFU treatment. Although coagulative necrosis was not detected in some lesions, the absent or significantly reduced tumor blood supply resulted in lesion shrinkage, which was also effective according to the RECIST1.1 criteria. At present, there is no universal HIFU standard that takes into account both types of efficacy evaluation. Therefore, this study formulated mRECIST1.1 combined with RECIST1.1, which is a tailored approach for clinical HIFU efficacy evaluation. This study exposed that despite most lesions showing varying degrees of coagulative necrosis after HIFU treatment, the remaining ones were stable or reduced in size, achieving a disease control rate of 90% at one month. Besides, the coagulative necrosis rate, DCR, and ORR were all higher in the Combined HIFU group compared with the Only HIFU group. In short, short-term efficacy was higher in the Combined HIFU group. Previous studies have established that the complete ablation rate of HIFU for HCC after a single treatment ranges between 28.5%-68% (33, 38, 49, 50). However, it was less than 10% in this study, ascribed to all subjects having advanced liver cancer, among which 64% (32/50) of cases were diagnosed with secondary liver cancer, and 54% (27/50) had lesions over 5 cm in diameter. Treatment goals were mainly palliative, potentially restraining treatment intensity. After a long-term follow-up period of 16.9 months, the median survival of patients with advanced liver cancer in this study was over 10 months, which was significantly higher than that of patients with untreated liver metastases (15). The marginally lower mOS in the Combined HIFU group compared to the Only HIFU group could be attributed to secondary liver cancer originating from different sites and multiple factors influencing long-term survival. A recent study indicates that HIFU significantly improves OS in CRC liver metastasis patients, but the number of metastases treated with HIFU and systemic treatment lines significantly influenced survival (51).

In terms of safety, Acute adverse events in this study consistent with the findings of earlier studies. No new or fatal adverse events occurred in the Combined HIFU group, the incidence of infectious fever and liver function impairment was significantly lower than that in the only HIFU group, which may be associated with antiangiogenic agents inhibiting tumor neovascularization and inflammatory factor release, normalizing abnormal blood vessels, minimizing focal blood supply, regulate the immune microenvironment, thereby reducing tissue damage and inflammatory responses (52, 53).

This study has drawn several preliminary conclusions of clinical value, but it also has its limitations. Specifically, the study was designed as a single-center, small-sample retrospective study and did not include key biological markers such as alpha-fetoprotein (AFP) and carcinoembryonic antigen (CEA). In this study, over 70% of the patients received other anti-tumor treatments after HIFU. The adverse events might be influenced by other factors and it was difficult to attribute them solely to HIFU. Therefore, only the analysis of acute adverse reactions was conducted, which might result in the omission of risks associated with late toxicity. Furthermore, the follow-up for long-term survival outcomes remains inadequate-for instance, specific subsequent treatment regimens were not incorporated into the study. In future research, a prospective large-sample stratified cohorts study will be conducted to address these limitations. This research approach is expected to provide more accurate conclusions for clinical practice, thereby offering evidence-based guidance for clinical decision-making and ultimately benefiting a larger patient population.

## 5 Conclusion

Antiangiogenic agents combined with HIFU are effective and safe in the treatment of advanced liver cancer and enhance the efficiency of HIFU treatment. Nonetheless, their efficacy and safety should be evaluated in large-scale prospective randomized clinical studies.

## Data Availability

The datasets presented in this article are not readily available because privacy restrictions. Requests to access the datasets should be directed to the corresponding author.
